# Effect of zinc sources and experimental conditions on zinc balance in growing wethers

**DOI:** 10.1093/tas/txac005

**Published:** 2022-01-12

**Authors:** Chanhee Lee, Jacob E Copelin, Mike T Socha

**Affiliations:** 1 Department of Animal Sciences, Ohio Agricultural Research and Development Center, The Ohio State University, Wooster, OH 44691, USA; 2 Zinpro Corporation, Eden Prairie, MN 55344, USA

**Keywords:** zinc amino acids, zinc glycinate, zinc hydroxychloride, zinc sulfate

## Abstract

Three experiments were conducted with growing wethers to evaluate apparent excretion and retention of Zn from various sources. In experiments 1 and 2, Zn-ethylene diamine (ZE), Zn hydroxychloride (ZHYD), Zn-lysine/glutamate (ZAA), and Zn-glycinate (ZG) were used and ZnSO_4_ (ZS), ZHYD, ZAA, and ZG were used in experiment 3. In experiment 1, eight wethers were used in a replicated 4 × 4 Latin square design. In experiments 2 and 3, 40 wethers were used in a randomized block design. In experiment 1, each period (total four periods) consisted of 14-d diet adaptation and 4 d of total collection of feces and urine. In experiments 2 and 3, wethers received a basal diet for 14 d and received experimental diets for 9 d (diet adaptation), followed by 4 d of total collection of feces and urine. Total collection was conducted in wooden metabolic cages. All data were analyzed using the MIXED procedure of SAS as a Latin square design for experiment 1 and a completed randomized block design for experiments 2 and 3. In all experiments, dry matter intake did not differ among treatments except that it tended to be different in experiment 2. In experiment 1, no difference in Zn excretion (88%) and retention (11%) as proportion of Zn intake was observed among Zn sources. In experiment 2, total tract digestibility of crude protein was greater (*P* < 0.01) for ZAA than ZE and ZG (82.0% vs. 79.1% and 77.8%, respectively) and greater (*P* < 0.01) for ZHYD than ZG (80.2% vs. 77.8%). However, total tract digestibility of neutral detergent fiber was low (on average 16%) for all treatments with no difference among treatments in experiment 2. Apparent excretion and retention of Zn as proportion of Zn intake did not differ among treatments, and Zn retention (~1.4% of Zn intake) was very low for all treatments. In experiment 3, ZHYD and ZAA had greater retention of Zn (17.8% vs. 1.5%; *P* = 0.01) than ZG. Fecal Zn excretion was greater (97.3% vs. 81.2%; *P* = 0.01) for ZG vs. ZHYD and ZAA, and Zn retention for ZG was only 1.5% of Zn intake. In conclusion, potential increases in Zn absorption and retention were observed for ZHYD and ZAA compared with ZS and ZG in experiment 3 and these differences were not found in experiments 1 and 2. Experiment 1 used a Latin square design and experiment 2 used a diet containing largely undigestible fiber. These experimental conditions may have affected Zn metabolism in wethers. Inconsistent results on Zn balance for ZG among the experiments warrant further studies regarding its bioavailability.

## INTRODUCTION

Zinc must be provided daily via a diet for optimum growth, production, and health due to its critical roles in metabolic functioning (NRC, 2001; NASEM, 2016). There are various forms of Zn available for ruminant animals and zinc sulfate is one traditional source, but alternate sources have been studied. Zinc sulfate is soluble in the rumen to some extent and the solubility of Zn (i.e., Zn ion dissociated) can negatively affect digestibility of other nutrients (Genther and Hansen, 2015) and may lower Zn absorption if it binds to rumen constituents (Caldera et al., 2019) or other metals (Goff, 2018). Organic Zn (complexed with various amino acids) or inorganic Zn in the form of Zn hydroxychloride have potential to increase Zn availability due to their relative inertness in the rumen (Spears, 1989; Shaeffer et al., 2017).

Zinc availability in experiments can be also affected by experimental factors such as experimental designs and basal diets (nutritional composition and quality). A Latin square and randomized complete block design are two popular designs used in ruminant nutritional research. Although both have been used in trace mineral research (Carmichael et al., 2019; Daniel et al., 2020), a randomized complete block design is probably preferred due to no carryover of treatment effects. If a carryover effect exists in a Latin square design, responses to trace mineral supply may differ between the two designs. Furthermore, it is well understood that Zn has antagonistic relationships with various minerals influencing Zn absorption (NRC, 2001; Goff, 2018), but also Zn absorption is likely affected by dietary undigestible fiber (Carmichael et al., 2019), which has not been well understood.

The objectives of the studies were to evaluate various sources of Zn on availability and examine potential experimental factors (experimental design and fiber digestibility) that influence Zn balance in wethers. We hypothesized that Zn balance is influenced by not only Zn sources but also experimental conditions.

## MATERIALS AND METHODS

The experiment was performed at Sheep Research Center at Agricultural Research and Development Center, The Ohio State University (Wooster, OH). All procedures involving animals and their care were approved by the Institutional Animal Care and Use Committee of The Ohio State University.

### Animals, Diets, and Procedures

In experiment 1, eight wethers (34 kg ± 1.8 SD; Suffolk × Dorset) were used in a replicated 4 × 4 Latin square design. The wethers were assigned to squares and wethers in each square were randomly assigned to dietary treatments, a basal diet supplemented with different sources of Zn: Zn ethylene diamine (ZE, Zinpro Corporation, Eden Prairie, MN); Zn-Glycinate (ZG; Pancosma, Rolle, Switzerland); Zn-lysine/glutamate complex (ZAA; Zinpro Corporation, Eden Prairie, MN); and Zn hydroxychloride (Zn_5_(OH)_8_Cl_2_·H_2_O; ZHYD; Micronutrients, Indianapolis, IN). The basal diet was prepared as a pelleted diet with no forage. Ingredients of the basal diet are shown in Table 1 and chemical compositions of the experimental diets are shown in Table 2. Premixes for different Zn sources were prepared with ground corn as a carrier (about 860 mg/kg on a dry matter [DM] basis) and the premixes replaced corn grain in the basal diet before pelleting to obtain the targeted dietary Zn concentration (80 mg/kg DM). The concentration of Zn in the four experimental diets were similar and about 52% of dietary Zn was provided from supplemental Zn. The diets were fed once daily for ad libitum intake with free access to water. Fourteen days were allowed for diet adaptation followed by 4 d of sample collection in each period. During the diet adaptation period, wethers were housed in group pens separated by dietary treatment (i.e., 2 animals per pen) and moved to wooden individual metabolic cages for total collection of feces and urine during the sample collection. All the wethers experienced the wooden cages for 2 d (6 h a day) for adaptation before starting the experiment. Daily feces were collected using a fecal bag attached to the wethers by a harness. Urine from wethers drained through slatted floor and was collected into a container under the cages. Feed offered and refused were monitored daily and samples of feeds and refusals were collected daily during the sample collection period. At the end of the experiment, feeds were composited by treatment and period and refusals were composted by wether and period. Total feces and urine were collected from individual wethers during the sampling period and daily subsamples of feces and urine from individual wethers were collected and composited proportionally by wether and period.

**Table 1.** Basal pelleted diet ingredients

Items	Exp. 1	Exp. 2	Exp. 3
		DM %	
Corn distillers grain	10.2		
Corn grain	60.0	42.0	28.3
Corn starch		3.9	3.9
Soybean meal	6.4		
Soycomil P^∗^		17.7	6.6
Soybean hulls	19.7		2.1
Corn cob		28.7	
Alfalfa meal			53.6
Animal/vegetable fat^†^	1.2	3.7	3.2
Urea	0.5		
Limestone	1.3	1.3	
Vitamin E	0.09	0.05	0.06
Vitamin A	0.01	0.01	0.01
Vitamin D	0.02	0.01	0.03
Bovatec 91^‡^	0.02	0.02	0.02
Selenium premix^||^	0.06	0.04	
Ammonium chloride	0.56	0.44	0.28
Copper sulfate	0.002	0.003	0.003
Manganese sulfate	0.01	0.008	
Potassium chloride		0.39	
Magnesium sulfate		0.39	0.25
Sodium bicarbonate		0.39	0.73
Calcium phosphate			0.77

ADM Alliance Nutrition, Inc., Quincy, IL.

G. A. Wintzer and Son Co., West Auglaize, OH.

Zoetis, Parsippany, NJ.

Selenium selenate, 200 mg/kg.

**Table 2.** Effects of Zn sources on Zn balance in a replicated Latin square design (experiment 1)

Items	Diets^∗^				SEM	*P*-value
	ZE	ZG	ZAA	ZHYD		
Chemical composition of diets (% of DM)						
DM, as-fed	89.0	89.2	88.9	89.1		
OM	95.6	95.7	95.6	95.7		
CP	16.4	16.9	16.7	16.6		
NDF	20.2	20.8	20.1	21.4		
ADF	12.3	12.5	12.5	12.9		
Basal Zn, mg/kg	39.5	39.5	39.5	39.5		
Total Zn, mg/kg	82.3	80.4	79.5	82.4		
*n*	8	8	8	8		
DMI, kg/d	1.8	1.8	1.7	1.7	0.07	0.64
Digestibility						
DM, %	78.2	77.8	78.7	77.8	0.67	0.71
OM, %	79.3	78.8	79.7	78.8	0.68	0.64
CP, %	72.2	72.7	73.2	72.2	1.10	0.49
NDF, %	45.9	47.0	49.2	47.5	2.25	0.62
Zn balance						
Intake, mg/d	147	143	138	142	6.69	0.62
Feces, mg/d	132	125	120	121	5.31	0.26
% of Zn intake	90.1	88.3	86.9	85.6	2.79	0.38
Urine, mg/d	1.33	1.49	1.47	1.23	0.256	0.65
% of Zn intake	0.92	1.07	1.05	0.86	0.172	0.42
Retained, mg/d	13.4	16.2	16.0	19.5	4.54	0.54
% of Zn intake	8.9	10.6	12.1	13.6	2.86	0.37

ZE, Zn ethylene diamine; ZHYD, Zn hydroxychloride; ZAA, Zn lysine/glutamate; ZG, Zn glycinate.

In experiment 2, 40 wethers (about 34 kg ± 3.0 SD; Suffolk × Dorset) were used in a randomized block design. Wethers were blocked by body weight (BW) into nine blocks with four wethers per block. The experiment consisted of three phases: phase 1, all animals received a basal diet without supplemental Zn (30 ppm on a DM basis) for 14 d; phase 2, all animals were housed in treatment pens and received the experimental diets containing different sources of Zn (ZE, ZG, ZAA, and ZHYD) for 9 d; phase 3, wethers were moved into the wooden metabolic crates and total feces and urine were collected for 4 d. Supplemental Zn premixes were prepared as in experiment 1 (about 800 mg/kg of Zn on a DM basis) and included in the experimental diets by replacing corn grain to reach the target dietary Zn concentration (65 mg/kg on a DM basis) before pelleting. Ingredients for the basal pelleted diet are shown in Table 1 and chemical compositions of the experimental diets are shown in Table 3. BWs of wethers were measured for two consecutive days at the beginning of phase 1 and end of phase 3. Samples of feeds, refusals, feces, and urine were collected for 4 d during phase 3 as in experiment 1. All wethers were fed once a day with free access to water.

**Table 3.** Effects of Zn sources on Zn balance in a randomized complete block design (experiment 2)

Items	Diets^∗^				SEM	*P*-value
	ZE	ZG	ZAA	ZHYD		
Chemical composition of diets (% DM)						
DM, as-fed	92.3	92.1	92.8	91.8		
OM	87.5	87.1	87.7	86.3		
CP	17.1	17.6	17.4	17.4		
NDF	29.2	29.9	28.7	28.4		
ADF	16.5	16.2	16.2	16.9		
Basal Zn, mg/kg	30	30	30	30		
Total Zn, mg/kg	111	72	69	66		
*n*	10	9	10	10		
DMI, kg/d	1.7	1.6	1.9	1.8	0.07	0.09
BW initial, kg	34.2	34.6	34.6	34.1	0.98	0.58
BW final, kg	45.1	45.5	45.8	45.4	0.80	0.53
Digestibility						
DM	65.3	63.7	65.6	65.5	0.81	0.17
OM	64.6	63.0	65.0	64.3	0.89	0.23
CP	79.1^bc^	77.8^c^	82.0^a^	80.2^ab^	0.82	< 0.01
NDF	16.5	17.9	15.8	14.6	48.35	0.99
Zn balance						
Intake, mg/d	193^a^	118^b^	128^b^	117^b^	5.1	<0.01
Feces, mg/d	187^a^	119^b^	120^b^	110^b^	6.6	<0.01
% of Zn intake	96.9	102.3	94.3	94.6	3.92	0.39
Urine, mg/d	1.92	2.14	1.65	2.21	0.23	0.30
% of Zn intake	1.01^c^	1.83^ab^	1.31^bc^	2.00^a^	0.20	<0.01
Retained, mg/d	3.97	−4.82	5.97	4.93	5.02	0.42
% of Zn intake	2.04	−4.13	4.44	3.36	3.94	0.36

ZE, Zn ethylene diamine; ZHYD, Zn hydroxychloride; ZAA, Zn lysine/glutamate; ZG, Zn glycinate.

Within row, least square means with uncommon superscripts differ, *P ≤* 0.05.

In experiment 3, 40 wethers (45 kg ± 7.0 SD; 32 Suffolk × Dorset and 8 Merino × Dorset) were used and the design and procedure of the experiment was the same as in experiment 2 except that wethers were blocked by breed and BW, ZnSO_4_ (ZS) was used instead of ZE, and ingredients in the basal diet were different as shown in Table 1. Chemical compositions of the experimental diets are shown in Table 4.

**Table 4.** Effects of supplemental Zn sources on Zn balance in a randomized complete block design (experiment 3)

	Diets^∗^				SEM	*P*-values
	ZS	ZG	ZAA	ZHYD		
Chemical composition of diets (% DM)						
DM, % as fed	91.2	91.2	91.5	91.2		
OM	92.4	92.3	92.4	92.4		
CP	17.5	17.6	17.4	17.9		
NDF	33.5	35.4	33.8	35.3		
Basal Zn, mg/kg	32	32	32	32		
Total Zn, mg/kg	89.2	75.5	81.8	82.7		
*n*	10	8	10	10		
DMI, kg/d	1.9	1.9	1.6	1.6	0.15	0.24
Initial BW, kg	45.5	44.8	44.6	45.1	2.33	0.29
Final BW, kg	56.9	56.8	55.9	55.8	2.37	0.50
Digestibility						
DM	65.6	65.4	66.8	67.0	1.05	0.62
OM	69.0	68.7	70.1	70.3	0.97	0.62
CP	72.6	72.3	73.1	75.0	0.97	0.25
NDF	41.0	44.1	41.1	44.4	2.31	0.53
Zn balance						
Intake, mg/d	171	146	134	133	13.2	0.11
Fecal Zn, mg/d	151^a^	141^ab^	111^b^	110^b^	12.6	0.05
% of Zn intake^†^	89.8^ab^	97.3^a^	81.5^b^	80.9^b^	3.93	0.01
Urine Zn, mg/d	1.93	1.74	1.19	1.60	0.368	0.45
% of Zn intake	1.05	1.22	0.89	1.15	0.158	0.50
Retained, mg/d	17.2	3.1	22.3	20.9	6.08	0.12
% of intake^‡^	9.1^ab^	1.5^b^	17.6^a^	18.0^a^	3.89	0.01

ZS, Zn-sulfate; ZHYD, Zn-hydroxychloride; ZAA, Zn-lysine/glutamate; ZG, Zn-glycinate.

ZS vs. ZHYD or ZS vs. ZAA, *P* = 0.08.

ZS vs. ZHYD or ZS vs. ZAA, *P* = 0.08.

Within row, least square means with uncommon superscripts differ, *P ≤* 0.05.

### Laboratory Assays

Feeds, refusals, and feces were sent to commercial labs (Rock River Laboratory, Watertown, WI for experiments 1 and 2 and Cumberland Valley Analytical Services, Waynesboro, PA for experiment 3) for DM, organic matter (OM), crude protein (CP), and neutral detergent fiber (NDF) wet-chemistry assays. The concentration of Zn in those samples were determined using inductively coupled plasma (ICP)-optical emission spectrometry after microwave digestion at STAR Lab (OARDC, The Ohio State University, Wooster, OH 44691; https://u.osu.edu/starlab). Urine samples were analyzed for Zn concentration using ICP-MS (Utah State University Veterinary Diagnostic Laboratory for experiments 1 and 2 and Iowa State University Veterinary Diagnostic Laboratory for experiment 3). Apparent total tract digestibility of nutrients (DM, OM, CP, and NDF) was calculated according to the following equation with an example with DM: Apparent DM digestibility (%) = 100 × (1 − [fecal DM excretion/{dietary DM fed − DM refused}]). The balance of Zn was calculated according to the following equation: Zn balance (mg/d) = dietary Zn fed − dietary Zn refused − fecal Zn excretion − urinary Zn excretion.

### Statistical Analyses

One wether on ZHYD in experiments 2 and two wethers on ZG in experiment 3 had fever and refused to eat the experimental diet during the 4-d collection period. Therefore, data from those wethers were not used in the following statistical analyses.

All data were analyzed using the MIXED procedure of SAS (SAS Inst. Inc., Cary, NC) as a Latin square design in experiment 1 and a completed randomized block design in experiments 2 and 3. Square and wether within square were random effects and treatment and period were fixed effects in experiment 1. Block and block by treatment were the random effects and treatment was the fixed effect in experiments 2 and 3. Statistical differences were declared at *P* ≤ 0.05. Differences between treatments with 0.05 < *P* ≤ 0.10 were considered as trends toward significance. Data were presented as least squares means.

## RESULTS

In experiment 1, nutrient composition was similar across the diets and total Zn concentration of the experimental diets were about 80 mg/kg (DM basis) and close to the formulated concentration (Table 2). About 50% of dietary Zn was provided from supplemental Zn sources. Zinc sources did not alter dry matter intake (DMI) of wethers and did not affect (*P* ≥ 0.49) total tract digestibility of nutrients. No difference in Zn intake and outputs (feces and urine) was found, resulting in no difference in Zn balance (average 11.3% of Zn intake) among different sources of Zn.

In experiment 2, nutrient composition was similar among the experimental diets and the target concentration of dietary Zn was 65 mg/kg (DM basis; Table 3). The diets of ZAA, ZHYD, and ZG had dietary Zn concentrations that were close to the formulated level, but ZE had about 70% greater Zn concentration (111 mg/kg) compared with the formulated level. An internal investigation indicated that the ZE diet was contaminated when prepared at the feed mill (OARDC). The mixer at the feed mill was used to mix a beef trace mineral premix high in Zn and was not cleaned before mixing the ZE diet. The source of Zn contaminating ZE was zinc sulfate so that the sources of Zn in the ZE treatment was a combination of ZS and ZE. For ZAA, ZHYD, and ZG, the proportion of supplemental Zn in dietary Zn was about 57%. During the experiment, Zn sources did not affect DMI and BW of wethers and did not alter total tract digestibility of DM, OM, and NDF. However, the digestibility of CP was greater for ZAA than ZG and ZE (82.0% vs. 79.1% and 77.8%, respectively; *P* < 0.01) and CP digestibility for ZG was lower than that for ZHYD. Daily intake and fecal excretion of Zn were greater (*P* < 0.01) for ZE compared with others with no difference among ZAA, ZHYD, and ZG. However, fecal Zn excretion as proportion of Zn intake did not differ among all treatments. Daily excretion of urinary Zn did not differ among treatments but that as proportion of Zn intake was greater (1.9% vs. 1.0%; *P* < 0.01) for ZHYD and ZG compared with ZE and was greater for ZHYD than ZAA. Daily Zn retained and that as proportion of Zn intake were not affected by Zn sources.

In experiment 3, dietary Zn concentration was similar to the target concentration (80 mg/kg on a DM basis) at formulation (Table 4). Therefore, the proportion of supplemental Zn in dietary Zn averaged 61%. The diets with different sources of Zn did not alter DMI and BW of wethers and did not affect total tract digestibility of DM, OM, CP, and NDF. Daily intake of Zn was not different (*P* = 0.11) among treatments but was numerically greatest for ZS followed by ZG, ZAA, and ZHYD. Fecal Zn excretion as percent of Zn intake was greater (*P* = 0.01) for ZG compared with ZAA and ZHYD. Fecal excretion as percent of Zn intake tended to be greater for ZnS than ZHYD or ZAA (*P* = 0.08; see the footnote). However, daily excretion of urinary Zn and that as percent of Zn intake did not differ among treatments. Therefore, Zn retained as percent of Zn intake tended to be greater (*P* = 0.08) for ZAA and ZHYD vs. ZS and was greater (*P* < 0.01) for ZAA and ZHYD vs. ZG.

## DISCUSSION

The original objective of the project was to evaluate absorption and retention of Zn from various Zn sources using growing wethers as a model for dairy cattle. However, through the experiments, we noticed that the Zn balance was affected by experimental conditions (design and basal diet), so we further examined effects of experimental factors on Zn balances as the secondary objective. The basal diets used in the experiments were formulated to be as similar as possible in nutrient composition to that of lactating dairy cows. However, the type of diets used was different (pelleted vs. total mixed ration) from a typical US dairy diet and the concentrations of some nutrients were different (i.e., NDF and forage NDF) as compared with a dairy diet. In addition, some feed ingredients used in the diets were not typical of those used in a US dairy diet (i.e., Soycomil P, corn cob, ammonium chloride, and Bovatec 91). Corn cob was used as the main fiber source in experiment 2 and Soycomil P and corn starch were used to provide a portion of the dietary protein and starch, respectively, in experiments 2 and 3. These ingredients were used to lower Zn concentrations in the basal diets. Therefore, although the experiments were conducted with wethers as a model for dairy cows to assess the absorption and utilization of Zn from different Zn sources, caution is needed to apply the results to dairy cows and further experiments with dairy cows need to be conducted to confirm the results. Furthermore, because wethers were used as a model for dairy cows, dietary Zn concentration was much greater than that recommended for sheep, which may have lowered dietary Zn absorption in the current experiments.

No effects of Zn sources on DMI and BW in the three experiments were consistent except that there was a tendency that DMI was lower for ZG compared with other sources in experiment 2. No effect of Zn sources on DMI and BW is in agreement with previous studies with sheep, beef cattle, and dairy cattle (Faulkner et al., 2017; VanValin et al., 2018; Carmichael et al., 2019), where various organic and inorganic Zn sources were examined. Alijani et al. (2020) observed an increase in DMI for Zn supplementation (ZnO, Zn-Met, and nano-ZnO) compared with no supplemental Zn, but no difference in DMI among Zn sources was found. This suggests that the level of Zn provided from various sources to wethers was not deficient in the current experiments.

Supplemental Zn (i.e., levels and sources) can affect rumen fermentation (Arelovich et al., 2000; Eryavuz and Dehority, 2009), altering total tract digestibility of nutrients. Faulkner and Weiss (2017) examined sulfate and hydroxychloride forms of Cu, Mn, and Zn with two fiber sources (forage vs. byproducts) in lactating cows, and found that the hydroxychloride forms of Cu, Mn, and Zn increased total tract NDF digestibility by 2% unit without affecting DM, OM, and CP digestibility. Similar results were observed in a study by Guimaraes et al. (2021) where steers fed a grass hay diet increased total tract digestibility of NDF and ADF and tended to increase DM and OM digestibility when hydroxychloride forms of Cu, Mn, and Zn were supplemented compared with sulfate forms. Miller et al. (2020) observed no effect of Zn, Mn and Cu source (sulfate vs. hydroxychloride) on portion of DM, OM, NDF, and starch digested in the total gastrointestinal tract of lactating cows fed either brown midrib or conventional corn silage. In that study, due to increased DMI of cows fed the hydroxychloride forms of Zn, Mn, and Cu, the researchers observed an increase in amounts of DM, OM, and NDF digested in the total gastrointestinal tract, but the increase in DMI did not translate into increased lactation performance. A study with sheep (VanValin et al., 2018) found no difference in NDF digestibility between sulfate and hydroxychloride forms of Zn, but the hydroxychloride form of Zn tended to increase DM, OM, and NDF digestibility compared with an organic form of Zn (Zn Met). In a study with steers comparing different sources of trace minerals including Zn (sulfate vs. hydroxychloride; Genther and Hansen, 2015), ruminal DM and NDF disappearance of a diet was determined using an in situ technique. In that study, DM disappearance was decreased with increasing supply of sulfate forms of trace minerals, but the decrease in DM digestibility did not occur with the hydroxychloride form of trace minerals. Although results are mixed among studies, excessive ruminal supply of Zn likely decreases fiber digestion and consequently DM digestibility (Eryavuz and Dehority, 2009). The effect of different Zn sources on rumen fiber digestion is likely due to differences in rumen solubility of Zn between Zn sources (Spears, 1996; Cao et al., 2000). In the current study, although two organic Zn sources that were soluble (ZG and ZAA) and an inorganic source that was insoluble (ZHYD) were examined, we observed no changes in total tract digestibility of DM and NDF. The lack of an effect of Zn sources on total tract digestibility of DM and NDF likely suggests that the supply of Zn was not excessive.

Dietary CP digestibility was affected by Zn sources only in experiment 2. The inconsistent effect of Zn source on dietary CP digestibility among the current experiments is difficult to explain. Supplementing a diet with Zn vs. no supplemental Zn has previously been shown to increase dietary N digestibility or retention in some studies (Carmichael et al., 2018, 2019; Alijani et al., 2020). However, the majority of studies showed no effect of Zn sources on N digestibility and retention (Pino and Heinrichs, 2016; Faulkner and Weiss, 2017; Alijani et al., 2020; Chen et al., 2020). Nevertheless, Daniel et al. (2020) observed that dietary CP digestibility decreased by a 1% unit when an organic form of Cu, Mn, and Zn (proteinate) replaced 30% of the inorganic form of these minerals. In our experiment 2, CP digestibility was greater for organic ZAA compared with organic ZE, but CP digestibility was higher for inorganic ZHYD than the organic ZG. However, those Zn sources did not affect CP digestibility in experiments 1 and 3. More studies are needed to better understand the relationship between Zn sources and protein metabolism.

Fecal excretion and retention of Zn were affected by the sources of Zn in experiment 3, but not in experiments 1 and 2. This led us to conclude that Zn utilization and excretion could be affected not only by sources of supplemental Zn but also by experimental conditions such as design or basal diets (see the discussion later). There are various factors that affect retention of dietary Zn and the major factor would be solubility, that is, ionization before absorption, ligand, and interaction with other molecules when ionized (Goff, 2018). According to the review by Goff (2018), results of studies assessing the availability of hydroxychloride or organic forms of trace minerals vs. sulfate forms are mixed (i.e., superior vs. similar), but overall the bioavailability of organic and hydroxychloride forms of trace minerals are likely superior to some degree (1.1–2 times) over sulfated forms. This is because hydroxychloride or organic forms of trace minerals remain inert in the rumen compared with sulfate forms resulting in less interaction with other antagonists. In the current experiments, we compared two inorganic Zn (sulfate and hydroxychloride) and three organic Zn (Lys/Glu, Gly, and ethylene diamine) sources. The tendency for decreased fecal Zn excretion (i.e., increased apparent absorption) and increased retention for ZHYD and ZAA compared with ZS in experiment 3 agrees with a study by Shaeffer et al. (2017) but not with others (Faulkner et al., 2017; VanValin et al., 2018). It should be noted that, in a study by VanValin et al. (2018) where lambs were fed diets with different Zn sources, the ZAA used was Zn-Met and the metabolizable Met supply was well in excess of the requirement due to both the protein content of the diet (19.6% CP) and the protein source fed (corn gluten meal). An in vitro work by Sauer et al. (2017) found that when an excess of the amino acid complexed with Zn was provided (i.e., excess Lys provided when assessing uptake of Zn from Zn-Lys), uptake of Zn by Caco-2 cells decreased. This was not observed when an excess of Lys, Met, and Glu was provided with zinc chloride.

No difference in excretion and retention of Zn between ZHYD and ZAA was observed in the current three experiments. A direct comparison of excretion (i.e., absorption) and retention between ZHYD and ZAA is scarce in ruminants, but one study by VanValin et al. (2018) found no difference between those sources in sheep. Although it was not different or numerically greater in experiment 1, urinary excretion as percent of Zn intake was statistically or numerically lower for ZAA than ZHYD in experiments 2 and 3, which agrees with results from Nockels et al. (1993) and Spears (1989) where inorganic sources of Zn and Cu were compared with AA complexes of Zn and Cu. If the different excretion of Zn (% of intake) observed in experiments 2 and 3 was true, the lower urinary excretion of Zn from AA complexes than inorganic sources may indicate that they are metabolized differently. The difference in metabolism between the inorganic hydroxy sources and the amino acid complex sources of trace minerals may lead to differences in performance of animals. For example, in a study by Perry et al. (2021), providing amino acids-complexed trace minerals (Cu, Mn, and Zn) to beef heifers tended to increase circulating pregnancy-associated glycoproteins on day 25 of post insemination and tended to improve embryo survival compared with inorganic trace minerals (hydroxychloride). In a study by Kerwin et al. (2021a, 2021b), feeding amino acid-complexed trace minerals (Zn, Mn, and Cu) to transitioning cows (−60 d before calving to 22 wk of lactation) increased milk yield until week 8 of lactation and average daily grain of calves and improved health status compared with hydroxychloride of trace minerals.

An interesting result was that fecal Zn excretion was greatest for ZG among Zn sources in experiment 3, resulting in lowest Zn absorbed and retained (close to 0% of Zn intake). In experiments 1 and 2, fecal Zn excretion as percent of Zn intake was numerically greater for ZG than ZHYD and ZAA. This suggests that Zn from ZG was less available than Zn from ZHYD and ZAA. Studies with ZG in ruminant animals are scarce. Alimohamady et al. (2019) compared different organic Zn sources (Zn-Met, Zn-proteinate, and ZG) with ZS in growing lambs. In these studies, dietary Zn digestibility and retention were not determined, and all organic Zn sources generally had positive effects on total tract digestibility of nutrients (e.g., DM and NDF) compared with ZS. However, the digestibility was lower for ZG compared with Zn-Met and Zn-proteinate with no difference between Zn-Met and Zn-proteinate, suggesting that ZG might be more soluble in the rumen and less available for absorption compared with other organic forms of Zn. In rats, Zn absorption (51% vs. 44%) and retention (33% vs. 25%) was greater for ZG vs. ZS (Schlegel and Windisch, 2006). There was no difference in apparent Zn digestibility when Zn-proteinate and ZS were compared with ZG in growing pigs (Paulicks et al., 2011). In contrast to our results, Spears et al. (2004) observed a tendency for apparent absorption and retention of Zn from ZG compared with Zn in sulfate or Met-complexed forms in beef steers. Based on the limited information, it is likely that ZG has similar or lower bioavailability of Zn compared with other organic Zn sources and has similar or greater bioavailability compared with ZS, which contrasts with our results in experiment 3. More studies are needed better understand ZG in ruminants.

Other factors may have affected the availability of Zn from ZG in the current study. Stress or disease alters Zn metabolism (Goff and Stabel, 1990) and previous research has shown that Zn absorption and retention decreased (negative Zn balance) when steer calves were exposed to stress from the combination of feed and water restriction and an injection of adrenocorticotropic hormone (Nockels et al., 1993). In the current study, restricted activities and movements with neck chains and isolation in the metabolic cages during the collection period in summer (all studies were conducted in June to August) may have been stressful for wethers. However, all the experimental wethers were under the same environmental conditions, and the stress during the total collection was not a factor for greater Zn excretion for ZG. Although Nockels et al. (1993) observed decreases in Zn absorption during stress, no difference in absorption was observed between different sources of Zn (ZS and Zn-Met). The lower retention of Zn from ZG as compared with ZAA might be true based on the theory that trace minerals bound to amino acids are absorbed into the enterocyte via amino acids transport system vs. transporters such as ZIP4 (Gao et al., 2014; Sauer et al., 2017; Zhang et al., 2017) and that Gly is less efficiently absorbed than other amino acids (Gardner, 1976; Reichl, 1989). Further research is needed to explore differences in Zn metabolism among Zn sources as affected by factors such as stress, animal type (monogastric vs. ruminant), and ligand.

The differences in Zn excretion and retention among sources that were observed in experiment 3 did not occur in experiments 1 and 2. Although Zn excretion and utilization were not statistically different in experiments 1 and 2, numerical differences among treatments agreed with those in experiment 3. For example, fecal excretion of Zn was lower, and retention was numerically greater for ZHYD and ZAA compared with ZG. The lack of a significant effect of Zn source on Zn absorption and retention in experiment 1 may indicate a limitation of a Latin square design to detect treatment differences in a trace mineral balance study (e.g., carryover effect). No effects of Zn sources on Zn excretion and utilization were observed in experiment 2 although the experimental design (i.e., randomized complete block design) was the same as in experiment 3. In experiment 2, Zn retention was close to 0% of Zn intake and similar among all treatments. A noticeable difference between experiments 2 and 3 is total tract apparent NDF digestibility. The digestibility of NDF in experiment 2 was substantially lower than in experiment 3 (16% vs. 43%). The low NDF digestibility in experiment 2 occurred due to corn cob being used as the major NDF source in the diet and its NDF digestibility is poor (27% of in situ 24-h NDF disappearance, Varga and Hoover, 1983). Carmichael et al. (2019) compared Zn metabolism between Zn supplementation regiments (inorganic + organic minerals containing Zn) and no Zn supplementation in steers fed diets differing in NDF content (36% vs. 23%). In that study, total tract NDF digestibility was lower (53% vs. 65%) for the low NDF diet compared with the high NDF diet and significant decreases in Zn absorption and retention and an increase in fecal Zn excretion (percent of Zn intake) were observed for the low fiber diet.

One potential explanation for low Zn retention with the low NDF digestibility diets is an increase in Zn binding to undigested fiber, resulting in increased fecal Zn excretion and decreased Zn absorption. This is a potentially valid hypothesis as Zn binds to fiber and the binding capacity of Zn is greater with indigestible than digestible fiber (Thompson and Weber, 1982). Another factor for the lower retention of Zn in diets with poor NDF digestibility could be due to lower availability of Zn from dietary ingredients. In experiments 2 and 3, about half of the dietary Zn was supplied from dietary ingredients. Kincaid and Cronrath (1983) found that roughly 30% of the Zn in alfalfa and grass hay was associated with the NDF fraction. Thus, the availability of Zn from feed ingredients would be low when fiber digestibility of feed ingredients is poor. However, Faulkner and Weiss (2017) and Faulkner et al. (2017) found no effect of fiber sources (forage vs. byproduct) on Zn absorption and retention in lactating cows. In that study, the forage-based diet contained 25% NDF and the byproduct-based diet contained 36% NDF (DM basis) and dietary NDF digestibility was 44.5% and 50.5%, respectively. The lack of effects of fiber sources on Zn metabolism may have been due to greater NDF digestibility in those studies. Further research is needed to better understand the interaction between Zn sources and dietary fiber digestibility on Zn metabolism.

One shortcoming of the current three experiments was that each experiment did have a treatment that provided no supplemental Zn (i.e., negative control) to allow an estimate of Zn retention from the dietary ingredients as well as the supplemental sources. The portion of Zn measured in each experiment is a composite of Zn retained from dietary feed ingredients as well as the supplemental Zn source. Previous research indicated that correcting for the portion of the supplemental trace mineral absorbed for the portion of trace mineral provided by dietary ingredients, based upon the retention of the trace mineral in the supplemented treatment either substantially increased the estimated retention of the supplemental Mn sources (6% to 16%; Weiss and Socha, 2005) or substantially decreased the estimated retention of the supplemental Zn sources (10% to 3%; VanValin et al., 2018). If in the present studies, we assumed that sheep were retaining 5% of the Zn provided by dietary ingredients, portion of ZG, ZAA, and ZHYD retained would be 17.2, 18.6, and 22.1 (experiment 1), −10.7, 4.2, and 3.4 (experiment 2), and 0.1, 24.8, and 22.6 (experiment 3), respectively.

In conclusion, we found evidence that different Zn sources alter Zn absorption and retention in growing wethers. However, the availability of Zn was influenced by experimental conditions (basal diet and experimental design) in addition to Zn sources. Zinc absorption and retention was greater for supplemental Zn from ZHYD and ZAA than Zn from ZG or ZS in the study with a randomized block design. The differences in Zn metabolism among sources were not statistically significant in the study with a Latin square design, suggesting that a randomized complete block design may be a more appropriate experimental design to evaluate a balance of trace minerals. The absorption and retention of Zn were very low (<5% of Zn intake) when dietary NDF was poorly digested regardless of Zn sources and may have been due to a large portion of the dietary Zn from both the Zn source as well as the Zn provided by dietary ingredients binding to undigested fiber. The glycinate form of Zn warrants further investigation as retention for this Zn source was low. Although the experiments used wethers as a model for dairy cows, similar studies with dairy cows are needed to confirm the results.
